# Xylanolytic *Bacillus* species for xylooligosaccharides production: a critical review

**DOI:** 10.1186/s40643-021-00369-3

**Published:** 2021-02-17

**Authors:** Rozina Rashid, Muhammad Sohail

**Affiliations:** 1 Department of Microbiology, University of Karachi, Karachi, 75270 Pakistan; 2 Department of Microbiology, University of Balochistan, Quetta, Pakistan

**Keywords:** *Bacillus*, Prebiotics, Xylanase, Xylooligosaccharides

## Abstract

The capacity of different *Bacillus* species to produce large amounts of extracellular enzymes and ability to ferment various substrates at a wide range of pH and temperature has placed them among the most promising hosts for the industrial production of many improved and novel products. The global interest in prebiotics, for example, xylooligosaccharides (XOs) is ever increasing, rousing the quest for various forms with expanded productivity. This article provides an overview of xylanase producing bacilli, with more emphasis on their capacity to be used in the production of the XOs, followed by the purification strategies, characteristics and application of XOs from bacilli. The large-scale production of XOs is carried out from a number of xylan-rich lignocellulosic materials by chemical or enzymatic hydrolysis followed by purification through chromatography, vacuum evaporation, solvent extraction or membrane separation methods. Utilization of XOs in the production of functional products as food ingredients brings well-being to individuals by improving defense system and eliminating pathogens. In addition to the effects related to health, a variety of other biological impacts have also been discussed.

## Introduction

Prebiotics are non-degradable ingredients or food supplements that can significantly impact the physiology of entire body and improve the health of host by specifically promoting the growth of intestinal bacteria (Gibson et al. 2010). In the light of existing health awareness, the interest in prebiotic food ingredients has been rising. According to an estimate by Grand View Research (2016), the worldwide prebiotic market will reach up to worth 7.11 billion $ by 2024. There are many compounds comprising a group of essential functional oligosaccharides with significant interest that have been studied for their prebiotic capabilities such as fructooligosaccharides (FOs) (Jain et al. 2014), galactooligosaccharides (GOs) (Marín-Manzano et al. 2013), pectic oligosaccharides (POs) (Míguez et al. 2016) cellooligosaccharides (COs) (Karnaouri et al. 2019) and xylooligosaccharides (XOs) (Mäkeläinen et al. 2009). As described by Yang et al. (2015), XOs promote the growth of probiotics, such as, *Bifidobacterium* spp. and prevent the proliferation of cancer cells in human colon (Le et al. 2020), therefore, these can be utilized in food and feed preparations. XOs are composed of 2–10 residues of β1 → 4-linked xylose and are converted to short chains fatty acids when fermented by probiotic bacteria.

The spore-forming *Bacillus* species are broadly used in the production of functional foods for their probiotic properties and food preservation potential. In various animal feed supplementation, *Bacillus* strains provide abundant benefits including improvement in intestinal microbiota, digestibility and immunomodulation (Bernardeau et al. 2017). The excellent fermentation potential of these strains along with enhanced product yield and total absence of harmful by-products, render them an excellent choice for industrial processes (Singh and Bajaj 2017). Additionally, their enhanced survivability under wrathful environment makes them more suitable candidates for the production of prebiotics (Elshaghabee et al. 2017). This review article summarizes various reports regarding XOs production by *Bacillus* spp. particularly by xylanolytic bacilli. The strategies used for separation and purification of XOs have also been discussed. Lastly, the health benefits conferred by XOs, in general, have been described.

## Prebiotics and their significance

Prebiotics are typically non-digestible compounds, therefore they become available to the gut microbes and serve as feed for the beneficial microflora. This stimulates the production of nutrients by the gut bacteria for the colon cells leading to a healthier digestive system (Davani-Davari et al. 2019). The mutual beneficial relationship between prokaryotes and the colon plays a key role in health and well-being which is why the potential of prebiotics is being explored (Aachary and Prapulla 2011; Olaimat et al. 2020). Studies suggest that anaerobic degradation of prebiotics leads to their fermentation by probiotics that stimulate colonization of host intestine by the probiotic bacteria (particularly, lactic acid bacteria). The fermentation of prebiotics also results in the release of short-chain fatty acids (SCFA) as by-products like acetate, butyrate and propionate. These SCFA may exert an anticarcinogenic effect because of the enhanced acid content in the colon, increased mineral absorption and by elaborating anti-allergic effect. They also promote the growth of *Lactobacillus* and *Bifidobacterium* spp. and restrict the growth of potential pathogenic species (Jain et al. 2014). Prebiotic ingestion is possibly obtained through the diet such as certain fruits and vegetables; however, the levels of the natural prebiotics are excessively low, signifying the need for increasing the levels of prebiotic intake (Míguez et al. 2016).

## Source and types of prebiotics

Prebiotics as natural components may be found in various food sources like milk, honey, sugarcane juice, fruits and vegetables such as onion, beans, legumes, Jerusalem artichoke, chicory, peas, leek, garlic, banana, rye and barley (Davani-Davari et al. 2019).

There are many types of prebiotics, amongst which FOs are widely described as naturally occurring oligosaccharides derived from natural inulin (Jain et al. 2014) and are found in asparagus, wheat, sugar beet, tomato, banana, garlic and chicory (Aachary and Prapulla 2011). GOs, the product of lactose extension, are obtained from lactulose. These generally stimulate *Lactobacillus* and *Bifidobacterium* spp. and are found in human and cow milk (Marín-Manzano et al. 2013). POs are derived from pectin and polydextrose constituted from glucose (Míguez et al. 2016). Pectin is plentiful in different agro-industrial bio-resources for example apple pomace, citrus peel, sugar beet and cranberry mash. These materials can hence be considered as a source of possible POs (Holck et al. 2014). COs derived from hydrolysis of cellulose, consist of a group of significant functional oligosaccharides with substantial interest as a potential material in food and chemical industries (Karnaouri et al. 2019). XOs constitute yet another promising prebiotic, found in some fruits, vegetables, wheat bran and bamboo shoot, some plant-based milks, and honey (Vázquez et al. 2000; Samanta et al. 2015). As reported by Jaskari et al. (1998) XOs can enhance in vitro growth of *Bifidobacterium* spp. more efficiently as compared to other oligosaccharides including FOs. In addition to the heat-resistant and acid-stable properties (Samanta et al. 2015), XOs have also been reported to be effective even when consumed in lower doses which support the preference of XOs over other types of prebiotics (Sako and Tanaka 2011).

## Xylooligosaccharides structure and their health benefits

XOs are the xylose sugar polymers produced from xylan component of plant fibers (Reque et al. 2019). Xylans are generally categorized into glucuronoxylan in hardwoods and arabinoxylan and glucuronoarabinoxylan in grasses. In arabinoxylans, the main (xylan) chain is substituted with α-arabinosyl residues. In case of glucuronoxylan, 4-O-methyl glucuronic acid is linked by α-(l → 2) linkages; while glucuronoarabinoxylans consist of backbones of 1,4-linked-β-d-xylose residues with heterogenous substitutions (Chakdar et al. 2016). The major products produced from the hydrolysis of xylan are xylose, xylobiose, xylotriose and xylotetraose with some residual oligosaccharides. The XOs with low degree of polymerization (2–10 monomers) are considered as potential non-digestible sugars, while those with ˂ 4 monomeric units encompass prebiotic applications because they encourage the beneficial bacteria in the human gut such as Bifidobacteria and inhibit the growth of pathogens (de Freitas et al. 2019). Initially, the role of XOs as food ingredient and their positive role for gastrointestinal health were explored in Japan (Kobayashi et al. 1991).

Lately, XOs (particularly xylobiose) has attracted interest as an effective prebiotic that has beneficial effects for animal and human digestion. XOs not only promote right type of commensals, but also help to improve structural components of gut (Finegold et al. 2014). Because of the acid stability properties and β-bonds present in the XOs, they are protected from degradation while passing through the stomach. It has been observed that linear XOs and arabino-xylooligosaccharides (AXOs) are fermented more quickly than oligosaccharides containing uronic acid (UXOs) (Kabel et al. 2002). Comparatively more intestinal bacteria can grow on XOs (Crittenden et al. 2002), whereas AXOs and UXOs can be utilized by fewer strains. Ohbuchi et al. (2009) reported that only few of the human fecal *Bifidobacterium* spp. could utilize UXOs as compared to XOs.

### Utilization of XOs by gut microbes as prebiotics

The gut-related effects of XOs are well documented in literature (Lin et al. 2016; Pan et al. 2019). One of the most significant features of XOs as food ingredients is their potential to enhance the growth of intestinal beneficial flora such as *Bifidobacterium* spp. (Yang et al. 2015). Research carried out in humans suggests the utilization of xylobiose by intestinal bacteria as it cannot be hydrolyzed either by saliva, gastric juice, pancreatin or intestinal mucosa (Grootaert et al. 2006). In vitro experiments provide evidence that *Bifidobacterium* spp. i.e. *B. infantis, B. longum* and *B. adolescentis* are capable to utilize both xylobiose and xylotriose, but prefer xylobiose as a major component (Okazaki et al. 1990). Suwa et al. (1999) also obtained enhanced growth of *Bifidobacterium* spp. in gastrointestinal tract by administration of XOs in rats. The utilization and break down of XOs are strain-specific, depending upon the DP present in XOs mixture. For instance, *B. bifidum* can effectively utilize XOs, while*,* some other intestinal microbes such as *Escherichia coli* and *Clostridium* spp. are unable to utilize XOs (Crittenden et al. 2002). However, *Bacteroides* can utilize XOs with lesser efficiency and most *Lactobacillus* species barely utilize XOs (Mäkeläinen et al. 2009). The exact mechanism of utilization of XOs by the gut microbiota is yet to be explored.

### Other biological effects of XOs

XOs demonstrate a variety of other biological effects which are unusual from the prebiotic activities and are related to modulation of gut including anti-oxidant and anticarcinogenic activities (Ando et al. 2004), blood glucose regulation (Lim et al. 2018) anti-allergic effects (Shimoda et al. 2011), anti-inflammatory and anti-infection properties, immunomodulatory action (Zhang et al. 2018) and cytotoxic activity (Chen et al. 2012). In addition to biological effects regarding human health, XOs have also been utilized in feed and for phyto-pharmaceutical (antibacterial agents against plant diseases) as well as plant growth regulating (Bhardwaj et al. 2019) purposes. The underlying mechanism and physiological relevance of these actions are not understood completely.

## Evaluation of prebiotic potential of XOs

In vitro prebiotic analysis of XOs by using human gut probiotics as a model system revealed maximum growth of *Lactobacillus brevis* in the presence of 0.5% XOs syrup (Geetha and Gunasekaran 2017). Fermentation of XOs by the well-characterized probiotic strains of *Bifidobacterium adolescentis* and *L. acidophilus* was used by Kallel et al. (2015) to evaluate the prebiotic effects of XOs produced from *B. mojavensis.* Another in vitro analysis also revealed that XOs enhanced the growth of probiotic bifidobacterial strains which completely utilized XOs with the production of beneficial short-chain fatty acids signifying their prebiotic function (Reddy and Krishnan 2016b)*.* Further studies on decomposition of XOs (xylobiose) in the gastrointestinal tract using synthetic model of digestive enzymes indicated that xylobiose was not hydrolyzed by the enzymes. However, studies in humans revealed that xylobiose was not excreted into urine or feces within 24 h of oral administration, indicating its decomposition by the gastrointestinal microbiota (Mäkeläinen et al. 2009). Mathew et al. (2018), also endorsed complete uptake of xylobiose and xylotriose by the probiotic bacteria *B. adolescentis* and *L. brevis.*

In a study, the digestibility of XOs by gastric juices and the effect of XOs on the bile acids absorption were compared with the effects of isomaltooligosaccharides (IOs) and FOs. HPLC analysis showed majority of the IOs and a part of the FOs were absorbed by the small intestinal juice, but XOs were not digested at all by any digestive enzymes. On account of the lower disaccharidase activity in the XOs group than in the other dietary oligosaccharide, sugar hydrolysis in the digestive tract may be hindered and blood glucose levels may be efficiently controlled by dietary XOs (Vázquez et al. 2000).

## Enzymes for the production of XOs

Xylanases are a widespread group of enzymes, involved in the production of xylose and XOs. Xylanase is a main-chain enzyme which randomly cleaves the *β*-1,4–glycosidic linkages in xylan (Subramaniyan and Prema 2002; Chakdar et al. 2016). Endo-β-1,4-xylanase (EC 3.2.1.8) is one of the noteworthy hydrolytic enzymes among various xylanases that de-polymerize xylan to xylobiose and xylooligomers. β-1,4-Glycosidic internal bonds in the polymer of xylan cleaved by these endo-β-1,4-xylanases while β-xylosidase (EC 3.2.1.37) is the main enzyme responsible for hydrolysis from non-reducing ends of xylooligosaccharides and xylobiose to liberate monosachharide (Moreira and Filho 2016). However, the side groups are removed by the catalytic action of α-L-arabinofuranosidases (EC 3.2.1.55), α-d-glucuronidases (EC 3.2.1.139), acetylxylanesterases (EC 3.1.1.72), ferulic acid esterases (EC 3.1.1.73) and p-coumaric acid esterases (EC 3.1.1.). However, most of the enzymes used for this purpose are recombinant (Liu et al. 2017) and are released extracellularly.

Nonetheless, the whole xylanolytic enzyme system along with accessory activities is required for the complete hydrolysis of xylan (Collins et al. 2005). Many bacteria and fungi have been reported for xylanase production (Sohail et al. 2009; Rehman et al. 2014; Naseeb et al. 2015; Walia et al. 2017; Shariq and Sohail 2018). However, bacteria are the chief producers of extracellular xylanase with higher yields (Nagar et al. 2013; Aarti et al. 2015). The large-scale cultivation of fungi or actinomycetes is comparatively less desirable due to their longer generation time, undesirable rheological changes and reduced oxygen transfer (Khusro et al. 2016). Bacterial genera, such as *Bacillus, Staphylococcus, Micrococcus, Cellulomonas, Paenibacillus, Pseudoxanthomonas, Arthrobacter, Rhodothermus* and *Microbacterium* have been reported to produce xylanases (Subramaniyan and Prema 2002; Chakdar et al. 2016). Though filamentous fungi have also been reported as a good source of xylanase, however, their enzymes suffer with the inability to withstand at higher temperature. Very few fungi like *Thermomyces lanuginosus* with dynamic thermostable xylanases producing capabilities have been reported with optimum growth temperature of up to 50 °C (Chadha et al. 2019) and half-lives of the enzymes at 70 °C. Although, xylanases from thermophilic eubacteria and archeae have significantly longer half-lives at 80 °C or higher temperatures than those from thermophilic fungi (Dahlberg et al. 1993). For instance, *B. amyloliquefaciens*, *B. halodurans* and *Bacillus* sp. JB 99 grow maximally at temperatures up to 70–80 °C (Breccia et al. 1998; Mamo et al. 2006; Shrinivas et al. 2010). However, *T. lanuginosus* is a source of recombinant GH11 xylanase at commercial scale which performs its catalytic function optimally at 75 °C (Chadha et al. 2019). Furthermore, fungi also co-produce cellulase that must be separated and removed, however, selective production of xylanase in *Trichoderma* and *Aspergillus* species is possible in presence of xylan as the sole carbon source (Selvarajan and Veena 2017). A few aerobic or anaerobic thermophilic fungi like *Thermomyces* spp. (Anand et al. 1990) and *Aspergillus* spp. are capable of producing xylanases only under acidic growth conditions while neutral or alkaline conditions found suitable for many thermophilic xylanolytic bacteria (Selvarajan and Veena 2017). There are few benefits of using thermostable and alkali stable enzymes in biotechnological processes including hemicelluloses saccharification and industrial pulping (Nakamura et al. 1993; Zeldes et al. 2015); primarily it enhances the reaction rate, promote higher mass transfer, gives enzymes longer half-life, reduces the chance of microbial contamination, improves the solubility of lignocellulosic substrates, eases the recovery of volatile products, and increases the enzymatic efficiency throughout industrial processes (Kumar and Satyanaana 2014; Yadav et al. 2018).

## Bacilli and their xylanolytic potential for the production of XOs

Bacilli are Gram positive, rod-shaped, aerobic or facultative anaerobic, spore-forming bacteria that belong to the genus *Bacillus*, phylum Firmicutes. These bacteria are frequently isolated from various environments including soil, air, water, vegetables, food and also from human and animal gut (Elshaghabee et al. 2017). The innate capacity to produce large amounts of extracellular enzymes, vitamins and antimicrobial compounds specifies the importance of *Bacillus* as major industrial workhorse. The world market for industrial enzymes is estimated to be 1.6 billion dollars, split between 20% food, 15% feed and 56% of general technical enzymes in which *Bacillus* spp. share about 50% of the market (Schallmey et al. 2004). *Bacillus* spp. are reportedly house of many industrially important enzymes such as amylase (Sohail et al. 2005), protease, lipase, and phytase from *B. subtilis*, and *B. amyloliquefaciens* (Bron et al. 1999), cellulases from *B. subtilis* (Deka et al. 2013), levansucrases from *B. circulans* (Perez Oseguera et al. 1996), chitinases from *B. thuringiensis* (Thamthiankul et al. 2001), cyclodextrin glycosyltransferase (CGTase) from *B. firmus* (Gimenez et al. 2019), tannase from *B. licheniformis* (Mondal et al. 2000) and esterases from *B. circulans* (Kademi et al. 2000). Additionally, *Bacillus* spp. have also been explored for the production of antimicrobial compounds, vitamins, carotenoids and prebiotics and for preservation of food. Among other microorganisms, *Bacillus* was found to be a potential source of xylanases (Table 1), due to its tolerance to high temperature and broad pH range such as *B. stearothermophilus, B. amyloliquefaciens* (Chakdar et al. 2016), *B. pumilus, B. halodurans* (Verma and Satyanarayana 2012), *B. subtilis*, *B. coagulans* and *B. circulans* (Schallmey et al. 2004; Bajpai 2014). Oakley et al. (2003) reported a type 2 xylanase (Xyn11X) from a thermophilic *B. subtilis* that exhibited thermostability for up to 343 K (69.8 °C). Another novel xylanase producer, *B. pumilus,* is reported to be used in pretreatment of unbleached oil palm soda-anthraquinone pulp (Bakri et al. 2016). *B. circulans* has also been reported to over-produce xylanase extracellularly under optimized condition (Selvarajan and Veena 2017).

**Table 1:** *Bacillus* species producing xylanases (IU mL^−1^) by utilizing different substrates

Strains	Substrate used	Xylanase (IU mL^−1^)	Strain type	Temperature °C	Reference
*B. subtilis*	Sugarcane bagasse	439.5	Wild	37	(Irfan et al. 2012)
*B. subtilis*	Wheat middlings	70.31	Wild	37	(Reque et al. 2019)
*B cereus*	Corn husk	2.20	Wild	65	(Ayishal Begam et al. 2015)
*B. tequilensis*	Wheat bran	41.30	Wild	80	(Kumar and Satyanaana 2014)
*B. licheniformis*	Commercial xylan	122.9	Recombinant	60	(Liu and Liu 2008)
*B. aestuarii*	Commercial xylan	0.18	Wild	60	(Chauhan et al. 2015)
*B. borstelensis*	Rice husk	6.81	Wild	60	(Budhathoki et al. 2011)
*B. circulans*	Oat spelt xylan	7.05	Wild	45	(Bocchini et al. 2007)
*B. amyloliquefaciens*	Brewer's spent grain	10.5	Wild	37	(Amore et al. 2015)
*B.* sp. *3A*	Locust bean gum	571.14	Wild	70	(Regmi et al. 2016)
*B. pumilus*	Oat spelt xylan	1,723	Wild	50	(Subramaniyan 2012)
*B. halodurans*	Cane molasses	69	Wild	80	(Kumar and Satyanaana 2014)
*B. mojavensis*	Agricultural waste	249.308	Wild	37	(Akhavan Sepahy et al. 2011)
*B. brevis*	Wheat straw	30	Recombinant	55	(Goswami et al. 2014)
*Bacillus* strains	Corn cob	180	Wild	30	(Avcioglu et al. 2005)

Xylanolytic enzymes are categorized in glycoside hydrolase (GH) families 5, 7, 8, 10, 11 and 43 (Lombard et al. 2014), although families GH10 and GH11 bear most of these enzymes (Collins et al. 2005). They vary in their mechanism of action, substrate specificity and structure (Motta et al. 2013). GH11 xylanases can hydrolyze the unsubstituted regions of arabinoxylan while GH10 xylanases cleave xylose linkages closer to side-chain arabinose residues. However, among the other families, GH8 xylanases act only on xylan while GH5, GH7, and GH43 xylanases also act as endoglucanases, arabinofuranosidases or licheninases (Collins et al. 2005). In a recent study, a novel XynA thermostable GH10 xylanase from *Bacillus* sp. KW1 was cloned and expressed. It exhibited a wide substrate spectrum with a good stability at high temperatures and a broad range of pH (6.0–11.0). It showed hydrolytic activities towards xylans as well as a wide variety of cellulosic substrates; such activities are not commonly found in other GH10 enzymes (Wang et al. 2019). Recently, novel endoxylanases from family GH30 specific for glucuronoxylan have been reported. These endoxylanases identify GlcA side chains of glucuronoxylan, and cleave the glycosidic β-1,4 linkages of the xylan backbone in an endo-specific mode as defined by the GlcA position (Maehara et al. 2018). The bacterial GH30 endoxylanases BsXyn30 from *B. subtilis* LC9 (Guo et al. 2018), XynC from *B. subtilis* (St. John et al. 2006), Xyn5B from *Bacillus* sp. strain BP-7 (Gallardo et al. 2010), XynA from *Erwinia chrysanthemi* (Vršanská et al. 2007) have been described to have a similar function as that of GH10 and GH11 xylanases for hydrolyzing lignocellulose but can also be applied in the baking industry (Guo et al. 2018). XOs produced by xylanases from Bacilli are discussed in detail in the section “Enzymatic production of XOs”.

## Strategies for XOs production

XOs can be produced at commercial scale by the hydrolysis of xylan component in hemicellulose of LC biomass (Quiñones et al. 2015). LC raw materials like bagasses, brans, corncobs, malt cakes, hardwoods and hulls are possibly available substrates for the production of XOs (Poletto et al. 2020). XOs production mainly consists of a two-step combination including the first step of fractionation of biomass components to obtain xylan and then its conversion to XOs either by chemical or enzymatic methods or a combination of both (Aachary and Prapulla 2011; Poletto et al. 2020) followed by a series of purification steps required for the higher yield (~ 70–95%) of XOs (Vázquez et al. 2000; Moure et al. 2006).

## Physicochemical methods for XOs production

Production of XOs through hydrolysis is carried out by degradation of xylan catalyzed by hydronium, with steam or water in a single step also known as hydro-thermolysis (Qing et al. 2013). Significant amount of XOs is produced during the deacetylation of xylan attached with acetyl or uronic acid groups making them more water soluble (Aachary and Prapulla 2011).

The chemical methods for the production of XOs require the use of diluted solutions of mineral acids or alkalis. The concentration and degree of polymerization (DP) of XOs produced by alkali or acid hydrolysis depends on the reaction time, temperature and concentration of the reagents. Chemicals like dilute H_2_SO_4_, strong alkali solutions (KOH, NaOH) or ammonia are generally used for XOs production. Although, this method does not require the use of corrosive chemicals to extract xylan, it necessitates more expensive and robust equipment that can be operated at high temperatures and pressure.

Autohydrolysis is a process catalyzed by acids naturally formed from biomass, such as acetic acid, or by adding other acids for the reaction to start. Autohydrolysis of corncob biomass at 150 °C was reported by Nabarlatz et al. (2004) to produce XOs in good yield. However, the use of chemicals and auto-hydrolytic methods may generate unwanted by-products, including toxic compounds such as hydroxymethylfurfural and furfural with uncontrolled DP (Wan Azelee et al. 2016) which may increase down-streaming cost. Therefore, the enzymatic process is considered more suitable (Kaprelyants et al. 2017), due to its efficiency and specificity towards the product with desired DP. Moreover, it does not produce undesired by-products engendering an easy and economical downstream process (Bian et al. 2014).

## Biocatalytic methods for XOs production using *Bacillus* spp.

The enzymatic production of XOs is accomplished by extraction of xylan and its hydrolysis by endoxylanase enzyme having little exo-xylanase and β-xylosidase activity in order to avoid the production of xylose. The use of milder xylan extraction strategies, for example, hydrothermal processes, can produce acetylated XOs which could have higher prebiotic proficiency than the non-acetylated counterparts (Arai et al. 2019). There are many literature reports available describing the enzymatic production of XOs from *Bacillus* spp. utilizing different substrates (Table 2), such as wheat middling (Reque et al. 2019), wheat bran (Zhao and Dong 2016), sugarcane bagasse (Brienzo et al. 2010; Bragatto et al. 2013), brewer spent grain (Amorim et al. 2019), beechwood (Freixo and De Pinho 2002), corncob (Chapla et al. 2012) and cotton stalks (Akpinar et al. 2007). In this process of oligosaccharide production, the enzyme can either be added directly to the reaction medium (Akpinar et al. 2010), or added in immobilized form (Milessi et al. 2016), or produced in situ by microbial fermentation (Dorta et al. 2006; de Oliva-Neto and Menão 2009).

**Table 2:** Xylooligosaccharides (XOs) yield from different *Bacillus* species

Microorganism	Substrate	XOs yield	Strain type	Reference
*B. mojavensis*	Garlic straw	29 ± 1.74%,	Wild	(Kallel et al. 2015)
*B. subtilis* 3610	Beechwood xylan	30.6 ± 0.4 mg g^−1^	Recombinant	(Amorim et al. 2019)
*B. pumilus*	Wheat bran	44.4%	Wild	(Geetha and Gunasekaran 2017)
*B. subtilis* KCX006	Ground nut oil-cake	48 mgg^−1^dw	Wild	(Reddy and Krishnan 2016a)
*B. subtilis*	Sugarcane bagasse	99%	Wild	(Reddy and Krishnan 2016b)
*B. subtilis*	Sugarcane bagasse	113 mgg^−1^	Recombinant	(Bragatto et al. 2013)

Since XOs production is carried out by xylanase, therefore production capacity of xylanases is needed to be amplified. More often, the XOs formed during growth is not recovered due to its conversion to xylose by β-xylosidase and subsequent bacterial metabolism. For the production of XOs, the enzyme complex should have low exo-xylanase or β-xylosidase activity to prevent the production of high amounts of xylose which has inhibitory effects on XOs production (Verma and Satyanarayana 2012; Jain et al. 2014; Chakdar et al. 2016). *Bacillus* strains have been reported to synthesize β-xylosidase-free endo-xylanase and other xylan-degrading enzymes by fermenting lignocellulosic (LC) materials leading to enhanced XOs production (Reddy and Krishnan 2016a).

In one study, simultaneous production of β-xylosidase-free endo-xylanase and XOs was reported from *B. subtilis* strain KCX006 under solid-state fermentation (SSF) of agro-wastes. Optimization of SSF conditions remarkably improved the xylanase and XOs production giving a final concentration of 3102 IU g^−1^ dw and 48 mg g^−1^ dw, respectively, from wheat bran and ground nut oil-cake substrates. The major products, xylobiose, xylotriose and xylotetraose, were purified from the culture supernatant by activated charcoal and then analyzed by HPLC (Reddy and Krishnan 2016a). In another study, a purified extracellular xylanase from *B. mojavensis* UEB-FK, showing considerable heat stability at 60 °C for 90 min and wide range of pH (3–9) was found to produce xylobiose and xylotriose by the hydrolysis of xylan extracted from garlic-straw (Kallel et al. 2015). In yet another study, a prebiotic syrup containing XOs and arabino-xylooligosaccharides with DP 4–10 obtained by the action of endoxylanase preparation of *B. subtilis* on wheat bran (US patent No. 20110020498). In a recent report by Mathew et al. (2018), XOs from a wild-type *Bacillus* strain (*Geobacillus stearothermophilus,* previously *B. stearothermophilus*) were produced by the hydrolysis of insoluble arabinoxylan component of pretreated wheat bran by endoxylanases*.* Other *Bacillus* strains like *B. mojavensis* A21 (Haddar et al. 2012) and *Bacillus aerophilus* KGJ2 (Gowdhaman et al. 2014) have also appeared to be efficient towards XOs (xylobiose and xylotriose) production by the action of alkaline xylanase after hydrolysis of corncob and commercial xylan, respectively. Another study on wild-type *B. subtilis* described the use of crude xylanase lacking β-xylosidase to produce extremely pure XOs (> 90%), mainly xylobiose, with insignificant concentration of xylose (0.4%) using ammonia-pretreated sugarcane bagasse (Reddy and Krishnan 2016b). Likewise, an endoxylanase (XylB) from *B. pumilus* B20 was purified using ammonium sulphate fractionation and ion-exchange chromatography and subsequently applied to produce short-chain XOs from the hydrolysis of xylan. The product was refined by ultrafiltration which recovered 44.4% of XOs with a DP of 2–5 or higher (Geetha and Gunasekaran 2017). Lin et al. (2011) compared the catalytic action of immobilized and free endoxylanases from *B. halodurans* for XOs production using corncob-xylan under the reaction conditions of pH 8.0, 50 °C, 2% of substrate and 12.8 Ug^−1^ xylan and time duration of 24 h. It was observed that the free enzyme was more efficient towards XOs production with DP ≥ 4 and 32.5% xylobiose and xylotriose; this indicated that free enzyme could act upon both, shorter and longer chains of xylan. In contrast, the immobilized form produced 25.2% of xylobiose and xylotriose suggesting that the immobilized enzyme was limited to shorter chain xylans.

Recombinant DNA technology along with the use of immobilized enzyme has also been employed to enhance XOs production. For instance, a recombinant endoxylanase, XynA, from *B. subtilis* immobilized on agarose glyoxal support released xylose-free xylobiose, xylotriose and xylotetraose from xylan and exhibited thermal stability (up to 56  °C); indeed, the immobilized enzyme was 8600-fold more stable than that of the free enzyme. The enzyme remained active even after recycling for 10 times and the yield remained unaffected (Milessi et al. 2016). More recently, beechwood xylan was used to produce XOs using *B. subtilis* 3610 wild type and its clones. The clone harbored *xyn2* gene from *Trichoderma reesei* containing endogenous secretion tag. The maximum yield of XOs per amount of xylan (306 ± 4 mg g^−1^) was obtained with a DP of 4–6 after 8 h of fermentation under optimum conditions of pH 6.0 and 42.5 °C, using 2.5 g L^−1^ of xylan at initial concentration that was increased up to 5.0 g L^−1^ at 3 h (Amorim et al. 2019).

## Purification strategies for XOs

For the production of food-grade XOs, main step is to refine the key product by separating the oligosaccharides of high molecular weight and sugars of low molecular weight that do not have beneficial properties. Various strategies have been employed for refining the crude liquors to eliminate undesired compounds and concentrate XOs as much as possible to achieve the necessary DP (Vázquez et al. 2000) with 75% to 95% yield. The prebiotic purification by the removal of polysaccharides and protein followed by ion exchange chromatography was reported by Broekaert et al. (2011). Practically, these methods are not considered economical for the large-scale production of XOs. However, membrane separation methods like ultrafiltration and nanofiltration are the preferred technologies for refining XOs owing to the high recovery rate without employing any solvent (Nabarlatz et al. 2007). Moreover, separation of XOs with different DP can readily be accomplished by ultrafiltration to remove oligosaccharides with undesirable DP (Wijaya et al. 2020) (Table 3). Nonetheless, the search for cost-effective method to separate XOs from hydrolysate has yet to be finished.

**Table 3:** Purification techniques documented in literature for recovery of xylooligosaccharides (XOs)

Product	Purification techniques	Substrate	pH and temperature	Recovery % (DP^*^)	Reference
Acidic XOs	Anion-exchange and size-exclusion chromatography	Birchwood xylan	50 °C	85 (NA)	(Christakopoulos et al. 2003)
Xylobiose and xylotriose	Nanofiltration	Corncob meal	pH 5.5, 55 °C	74.5 (< 5)	(Yuan et al. 2004)
XOs	Gel filtration chromatography	Oil palm empty fruit bunch	NA	83–85 (5–40)	(Ho et al. 2014)
XOs	Ultrafiltration	Almond shells	179 °C	58.3 (NA)	(Nabarlatz et al. 2007)
Xylobiose	Nanofiltration	Empty fruit bunch	pH 5,50 °C	90.1 (NA)	(Wijaya et al. 2020)
XOs	Ultrafiltration	Wheat bran	pH 6.5, 60 °C	44.4 (2–5)	(Geetha and Gunasekaran 2017)
Xylohexose Xylobiose and xylotriose	High-performance anion exchange	Chemical pulp	pH 5,50 °C	47 & 90.5 (NA)	(Wang et al. 2018)
XOs	HPLC	Corncob	pH 8, 50 °C	32.5 (≤ 4)	(Lin et al. 2011)

DP^*^ degree of polymerization, *NA* not available

## Solvent extraction

Solvent extraction is often employed to recover XOs and remove pre-extraction interfering compounds arise from autohydrolysis mixers. Vacuum evaporation is initially applied to extract the unrefined XOs solution generated by hydrothermal processing and to remove potential explosives (Qing et al. 2013). Solvent extraction is helpful for evacuating non-saccharide segments, yielding both, a specifically refined liquid phase and a solvent portion which comprised phenolics and extractive-derived residues (Moure et al. 2006). However, the extent of purification and yield depend upon the solvent applied for extraction and the type of LC materials. Acetone, ethanol and 2-propanol are the most common choices to purify XOs solution. Studies have revealed that the precipitation of hemicellulose-derived products is often hindered due to the presence of water, even when present in very minute quantities; therefore, solvent extraction of freeze-dried solids resulted in the product of high purity using ethanol but with very low recovery (Vegas et al. 2005).

## Adsorption

Adsorption has been applied to separate XOs or to remove the unwanted compounds. It is usually coupled with other refining steps utilizing the most widely used adsorbents, acid clay, activated charcoal, diatomaceous earth, titanium, bentonite, silica, aluminum oxide and other synthetic materials (Xu et al. 2019). However, activated charcoal remained the most popular adsorbent, for instance, in the work by Pellerin et al. (1991) XOs were first retained by activated charcoal followed by elution with different concentration of ethanol. Reddy and Krishnan (2016a) employed the same method using activated charcoal to recover XOs produced by *B. subtilis.* The culture extract was treated with 10% w/v activated charcoal which was retained by vacuum filtration. The XOs adsorbed on top of charcoal (charcoal-XOs cake) were then eluted with 15%, 30% and 90% ethanol leading to molecular weight-based fractionation of XOs.

## Chromatographic purification

This method is used to fractionate XOs with high purity at analytical level by employing high-performance liquid chromatography (HPLC), ion-exchange, affinity and size-exclusion chromatographic separation techniques (Geetha and Gunasekaran 2017). Reddy and Krishnan (2016b) purified xylobiose through HPLC analysis, produced by the action of crude enzyme from *B. subtilis*. In another study, the chromatographic purification of XOs from autohydrolysis of oil palm empty fruit bunches resulted in a product with 74–78% purity, 83–85% of which was XOs (Ho et al. 2014). These methods have limited application to purify the samples prior to structural characterization of XOs by nano-spray mass spectrometry or ^13^C NMR (Moure et al. 2006).

## Membrane filtration techniques

Membrane separation technology, chiefly nanofiltration and ultrafiltration, are gaining interest at industrial level for many purposes including fractionation of high-purity XOs (Qi et al. 2012). Nanofiltration concentrates XOs and removes low molecular weight substances like phenolic compounds and monosaccharides (Ko et al. 2013) while separation of XOs from high molecular weight compounds with different DP is facilitated by ultrafiltration (Nabarlatz et al. 2007). In contrast to the other purification techniques mentioned above, membranes have various points of interest including low energy prerequisites, effective control of basic operational factors, and relatively simple scale-up (Cano and Palet 2007). Nanofiltration and ultrafiltration methods were used by Yuan et al. (2004) and Geetha and Gunasekaran (2017) who recovered 74.5% and 44.4% XOs produced by *Bacillus* sp., respectively. However, membrane filtration method can be influenced by structural properties of oligosaccharides, including the type of monosaccharides, linkages and substitutions in the structure of oligomer and oligosaccharides’ solubility (Pinelo et al. 2009).

## Applications of XOs

**Fig. 1:**
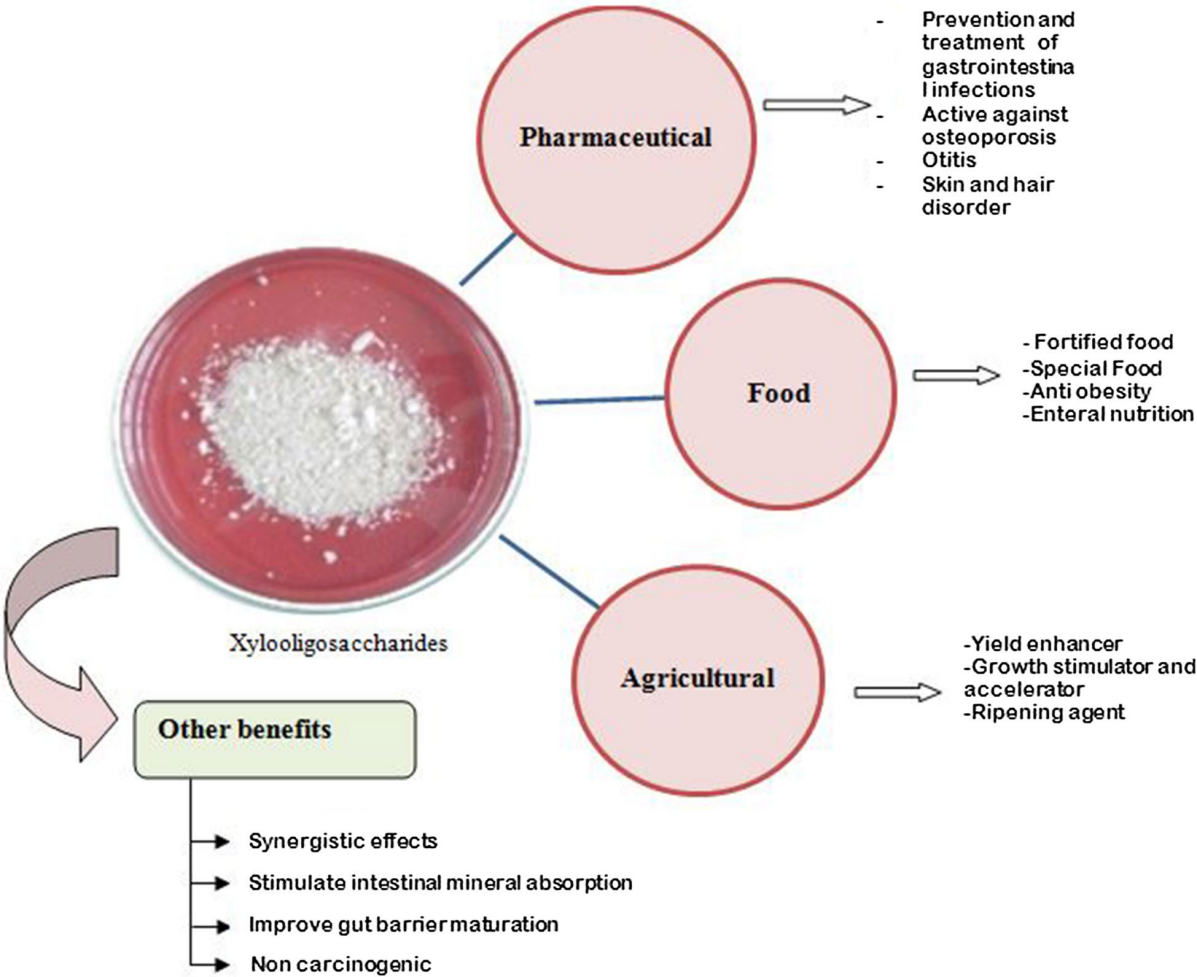
Applications of xylooligosaccharides (XOs)

XOs have an excellent potential for a variety of applications such as food industries, pharmaceutical and agriculture industries (Fig. 1). Currently the most significant applications of XOs based on the market demand correspond to functional food ingredients, for example nutritive preparations, as supplements in yogurt, soymilk, cocoa drinks, tea, jam, jellies, dairy products, candies, pastries, cakes, puddings, biscuits and fortified food for kids and adults. Due to the prebiotic characteristics of XOs, synbiotic nourishment foods (combination of prebiotic and a probiotic) have also been manufactured (Jagtap et al. 2017). The details of data available in literature regarding applications of XOs in various food and pharmaceuticals are presented in Table 4.

**Table 4:** Use of xylooligosaccharides in different fields

Category	Use of XOs	Documented in
Pharmaceutical application	Anti-allergic activity by producing inhibitory compounds against IgE antibody. Also has some anti-oxidant activity	Synthesis of xylooligosaccharides of daidzein and their anti-oxidant and anti-allergic activities (Shimoda et al. 2011)
	It was observed in vitro that XOs has some cytotoxicity towards leukemia cells obtained from acute lymphoblastic leukemia	Hot-compressed-water decomposed products from bamboo manifest a selective cytotoxicity against acute lymphoblastic leukemia cells (Ando et al. 2004)
	XO consumption has been found to significantly reduce severe constipation in pregnant women with no side effects	Effect of xylooligosaccharide intake on severe constipation in pregnant women (Tateyama et al. 2005)
	XOs as sole carbon source remarkably promote the growth of probiotics. It can be employed in food related applications as it has excellent anti-oxidant activity	Xylooligosaccharides production by crude microbial enzymes from agricultural waste without prior treatment and their potential application as nutraceuticals (Jagtap et al. 2017)
	Oral administration of Rice husk supplemented with XOs has beneficial, antidiabetic potential and significantly regulates blood glucose, insulin resistance and dyslipidemia by maintaining gut microbiota	Antihyperglycemic effect of rice husk derived xylooligosaccharides in high‐fat diet and low‐dose streptozotocin‐induced type 2 diabetic rat model (Khat-udomkiri et al. 2020)
Novel application	Esters and ethers have been manufactured from XOs (with high molar mass) utilized in biodegradable plastics as thermoplastic compounds. They may be used in coatings and films in food industry as well as capsules and tablets coatings	Thermoplastic pentosan-rich polysaccharides from biomass (Glasser et al. 1995)
	XOs can be used for the preparation of hydrogels	Preparation and properties of hydrogels based on hemicelluloses (Gabrielii and Gatenholm 1998)
Novel food	It is declared by the European food safety authority (EFSA) Panel on Dietetic Products, Nutrition and Allergies (NDA) that XOS is safe to be used in novel food (NF) such as dairy products, bakery products, chocolates, fruit jellies, and drinks	Safety of xylo-oligosaccharides (XOS) as a novel food pursuant to Regulation (EU) 2015/2283 By European food safety authority (EFSA) (Turck et al. 2018)
Food application	The synbiotic soymilk supplemented with XOs inoculated with a probiotic revealed anticarcinogenic effects with significant increase in functional compounds and nutrients by fermentation process	Synbiotic fermented soymilk with Weissella cibaria FB069 and xylooligosaccharides prevents proliferation in human colon cancer cells (Le et al. 2020)
	Xylobiose supplement is an alternative sweetener, exhibits therapeutic capacity for the treatment of metabolic disorders in obesity as well as suppression of fat deposit, reduces body weight, blood glucose, liver weights and blood lipids	Xylobiose prevents high-fat diet-induced mice obesity by suppressing mesenteric fat deposition and metabolic dysregulation (Lim et al. 2018)

## Concluding remarks

*Bacillus* species have secured their position as predominant bacteria in large-scale microbial fermentations as they exhibit various desirable properties. These species have the entire earmark of being a wellspring of xylanolytic enzymes and their utilization for bioprocessing of LC biomass could be considered a promising source for prebiotic and other value-added products. There are a number of works explained in this review that showed xylanolytic enzymes from Bacilli can reduce the DP of extracted XOs and increase the yields of oligomers which can be a significant aspect from the economic point of view. XOs with a low DP have incredible potential to stimulate and proliferate the intestinal microflora. The studies have indicated effective utilization of low DP XOs by probiotic *Bifidobacterium* and *Lactobacillus* spp. that further provide various other health benefits such as by producing short-chain fatty acids.

The large-scale purification of XOs from polysaccharides and monosaccharides is bottle neck to achieve cost-effective production of these prebiotics. Membrane filtration and adsorption techniques have greatly been employed to purify XOs than that of chromatographic methods. Advancement in membrane filters may lead to the development of cost-effective purification process.

XOs along with probiotics can be used to manufacture functional and novel food having the capacity to minimize gastrointestinal diseases, cancer, diabetes and obesity, thus leading to better health. Synbiotics may be an encouraging approach for future research by exploring the advanced knowledge of the symbiotic associations between XOs, gut microbiota and physiopathology of the whole body. However, more investigations for developing feasible and economical technologies to reduce the production cost, promoting enhanced xylanase production followed by high-purity XOs production and recovery are required.

## Data Availability

Data set was not generated during writing this review article and data sharing is not applicable.

## Abbreviations

AXOs: Arabinoxylooligosaccharides
COs: Cellooligosaccharides
DP: Degree of polymerization
EC: Enzyme commission
FOs: Fructooligosaccharides
GH: Glycoside hydrolase
GOs: Glactooligosaccharides
HPLC: High-performance liquid chromatography
LC: Lignocellulosic
NMR: Nuclear magnetic resonance
POs: Pecticoligosaccharides
SCFA: Short-chain fatty acids
SSF: Solid-state fermentation
XOs: Xylooligosaccharides
